# A microfluidic renal proximal tubule with active reabsorptive function

**DOI:** 10.1371/journal.pone.0184330

**Published:** 2017-10-11

**Authors:** Else M. Vedula, José Luis Alonso, M. Amin Arnaout, Joseph L. Charest

**Affiliations:** 1 Biomedical Microsystems Group, Draper, Cambridge, Massachusetts, United States of America; 2 Leukocyte Biology and Inflammation Program, Department of Medicine, Nephrology Division, Massachusetts General Hospital and Harvard Medical School, Charlestown, Massachusetts, United States of America; George Washington University School of Medicine and Health Sciences, UNITED STATES

## Abstract

In the kidney, the renal proximal tubule (PT) reabsorbs solutes into the peritubular capillaries through active transport. Here, we replicate this reabsorptive function *in vitro* by engineering a microfluidic PT. The microfluidic PT architecture comprises a porous membrane with user-defined submicron surface topography separating two microchannels representing a PT filtrate lumen and a peritubular capillary lumen. Human PT epithelial cells and microvascular endothelial cells in respective microchannels created a PT-like reabsorptive barrier. Co-culturing epithelial and endothelial cells in the microfluidic architecture enhanced viability, metabolic activity, and compactness of the epithelial layer. The resulting tissue expressed tight junctions, kidney-specific morphology, and polarized expression of kidney markers. The microfluidic PT actively performed sodium-coupled glucose transport, which could be modulated by administration of a sodium-transport inhibiting drug. The microfluidic PT reproduces human physiology at the cellular and tissue levels, and measurable tissue function which can quantify kidney pharmaceutical efficacy and toxicity.

## Introduction

The human kidney regulates solute and water homeostasis, excretes metabolic waste, produces hormones, and maintains acid-base balance [1]. The nephron, the basic structural and functional unit of the kidney, has two key segments: the glomerulus, which filters the blood, and the tubule, which reabsorbs components of the filtrate into the peritubular capillary network. The proximal tubule (PT) segment reabsorbs the bulk of the filtered load into the bloodstream driven mainly by active sodium transport, while distal and collecting duct segments fine-tune the composition of the remaining filtrate to maintain volume and tonicity. Diabetes mellitus, high blood pressure, autoimmunity, off-target effects of certain drugs, or other insults generate defects in the glomerulus or PT, impairing the crucial kidney functions of filtration and/or reabsorption. These defects present clinically as loss of proteins, impaired metabolic and ionic homeostasis, and decreased clearance, potentially leading to advanced kidney failure.

In the PT, human renal proximal tubule epithelial cells (hRPTEC) form a polarized epithelial barrier enabled by cell-cell tight junctions (TJs). The main function of the barrier is selective reabsorption, largely dictated by the sodium-potassium adenosine triphosphatase (Na^+^/K^+^-ATPase) of the hRPTEC. An underlying basement membrane supports hRPTEC tissue, supplies chemical and mechanical signals, and separates the tissue from interstitial space and surrounding microvasculature. Generally, cells integrate various external signals including extracellular matrix (ECM) components, mechanical stimulation, and soluble signals from adjacent and distant cells. Tissue structure formation depends on critical information from the extracellular microenvironment to cue tissue orientation, energy metabolism and differentiation. The complex reabsorptive function of the kidney directly results from the specific tissue structure formation of the proximal tubule and surrounding peritubular microvasculature[2,3,4]. Therefore, an *in vitro* model that replicates the PT microenvironment will enable physiological tissue structure formation and direct quantification of renal reabsorptive function.

Advanced *in vitro* PT kidney models have incorporated physiological signals into the culture environment, such as fluid flow, ECM proteins and multiple cell types[5,6,7]. However, physiological reabsorption in the proximal tubule takes place across opposing monolayers of epithelium and endothelium separated by a basement membrane (BM), which is not replicated in these advanced models. Co-incubation of endothelial and epithelial cells in culture dishes show altered ECM expression and transport proteins in hRPTEC [8,9], which highlights the importance of the endothelial-epithelial co-culture. A PT model comprising epithelial and endothelial co-culture opposing each other in a physiological orientation with access to controlled levels of flow-induced shear stress (FSS), BM topography and tissue-probing metrics, represents a significant improvement in kidney models and may allow direct measurement of reabsorptive transport function *in vitro*.

**Fig 1:**
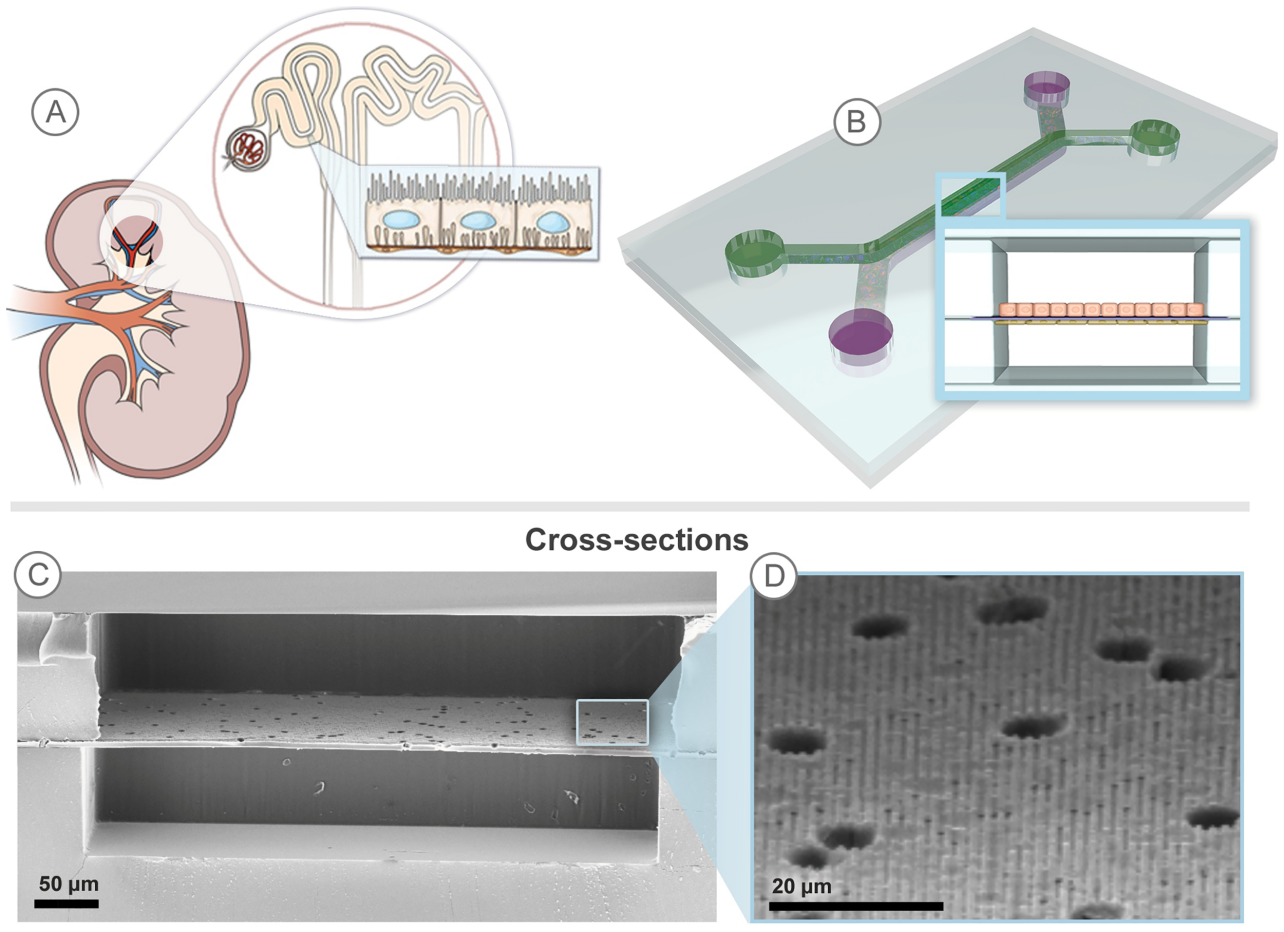
An in vitro 3D microfluidic model mimics the reabsorptive barrier of the proximal tubule. A) In vivo, water and solutes cross an epithelial-endothelial barrier in the reabsorption process from filtrate tubule to the peritubular capillaries. (B) The microfluidic channels overlap to create a filtrate channel (green) in communication with a vascular channel (purple). The cross-sectional architecture (inset) mimics in vivo epithelial-endothelial barrier and generates cell-mediated transport through the membrane. (C) A cross-sectional SEM of the device shows a semi-porous membrane, which serves as a scaffold for the epithelial and endothelial cells and separates the filtrate and vascular channels. (D) The membrane sub-micron ridge/groove topography influences tissue organization and function.

We present an *in vitro* three-dimensional microfluidic model of the (PT) reabsorption barrier designed to allow precise control over the microenvironment, physiological tissue structure formation, and direct evaluation of renal-specific reabsorptive function. We developed a co-culture of an hRPTEC layer opposing a human microvascular endothelial cell (hMVEC) layer in a microfluidic PT, mimicking the architectural design of the renal proximal tubule (Fig 1A and 1B). The presence of hMVEC in the microfluidic PT increased hRPTEC proliferation such that the hRPTEC count was more than double after 7 days of culture compared to single cultures of hRPTEC. Tissue layers were more compact with higher mitochondrial activity per cell and hRPTEC viability was supported for at least two weeks in co-culture. The microfluidic PT expressed kidney-specific transport proteins, the sodium-glucose linked transporter-2 (SGLT2), the Na^+^/K^+^-ATPase, and aquaporin 1 (AQP1), required for transepithelial water and ion transport. The microfluidic PT demonstrated inhibition and recovery of active reabsorption of a fluorescent glucose analog, 2-(N-(7-nitrobenz-2-oxa-1,3-diazol-4-yl)amino)-2-deoxyglucose (2-NBDG), in response to modulation of the drug ouabain. The model replicates both the structure and active function of the *in vivo* PT reabsorptive barrier, thus providing a platform with a functional metric to study the response of tissue to external stimuli, tissue structure development, transport mechanisms, and PT injury.

## Materials and methods

### Microfluidic PT device assembly

Polycarbonate track-etched membranes were hot embossed with sub-micron surface topography and assembled into a multilayer microfluidic device, described previously [10]. Briefly, the membrane was bonded to each channel layer with an RTV silicone adhesive (Dow Corning, Midland, MI) such that the patterned side of the membrane served as the floor to one of the cell chambers. The bottom microfluidic channel layer was then oxygen plasma bonded to a glass coverslip. Devices with incomplete bonding or irregular channel geometry were discarded prior to cell culture.

### Cell culture and seeding cells in the device

Normal-human microvascular endothelial cells (Lonza, CC-2543) expressed alpha actin and were tested negative for mycoplasma in compliance with Lonza’s Quality System. hMVEC were passaged twice prior to seeding them in the device and were maintained in hMVEC-complete media (growth media and supplements, Lonza, CC-3202) containing supplements of human epithelial growth factor (hEGF), hydrocortisone, 5% FBS, VEGF, hFGF-B, R^3^-IGF-1, ascorbic acid and GA-1000 (gentamycin, Amphotericin-B). Human renal proximal epithelial cells (hRPTEC) (ScienCell, #4100) were characterized according to ScienCell Quality Control and demonstrated expression of cytokeratin-18, -19 and vimentin and were negative for mycoplasma DNA measured by PCR. hRPTEC were passaged once prior to seeding them in the device and were maintained in hRPTEC complete media, a DMEM-F12 base supplemented with 0.5% FBS, 10ng/ml hEGF, 5 μg/ml insulin, 0.5 μg/ml hydrocortisone, 0.5 μg/ml epinephrine, 6.5 ng/ml Tri-idothyronine, 10 μg/ml transferrin, 100 U/ml penicillin, and 100 μg/ml streptomycin. Media was changed every other day until passage or seeding. Cultured cells were kept in a humidified 37°C incubator at 5% CO_2_. Bilayer microfluidic devices were chosen such that the topography on the membrane served as the cell culture surface in the epithelial channel. Devices were plasma treated using an oxygen plasma asher to render the channels hydrophilic and sterilized in 70% ethanol for 30 minutes. Channels were thoroughly rinsed in PBS and then incubated with a 60 μg/ml collagen type IV (from human placenta, Sigma-Aldrich, C-5533) solution for 2 hours at room temperature. The devices were again thoroughly rinsed in PBS before being prepped for hMVEC seeding. hMVEC were seeded into the vascular channel, and after attachment, the cells were allowed to proliferate to confluency over 3 days on the surface of the membrane. hRPTEC were then seeded into the filtrate channel and grew to confluency over 3–4 days, with both cell types in a common media of hMVEC-complete media. Confluency of the cell layers was evaluated by phase-contrast microscopy for each device before using it for experiments. Media was exchanged in both filtrate and vascular channels every day without applying shear stress for significant amounts of time until devices were used for experiments at day 7–14. Tissue samples were excluded from experiments if incomplete monolayers were observed prior to testing via phase contrast imaging. Complete tissue layers were randomly selected for each experimental group.

### Immunofluorescent labeling

Both hRPTEC and hMVEC were fluorescently labeled with antibodies directed against specific markers for differentiated epithelia, endothelium, and transport proteins. First, cells in devices were rinsed gently with PBS. For each rinsing, fixing, permeabilizing and labeling step, 100–150 μl of the solution was pipetted on top of the device to form a bubble on the surface. A micropipette was used to gently withdraw the solution through the channels several times. Cells were fixed with a 3.7% paraformaldehyde solution for 10 minutes at room temperature. Channels were rinsed with PBS 3 times and permeabilized with a 0.1% triton-x solution for 5 minutes at room temperature. Channels were again rinsed 3 times with PBS and blocked with a 1% solution of FBS for 3 hours. Primary antibodies were mixed at the appropriate concentration in a 1% FBS solution. A mouse anti-ZO1 (BD biosciences, 610967) and a rabbit anti-Von Willebrand Factor (vWF) (Abcam, ab9378) were used to label epithelial cells and endothelial cells, respectively. Primary antibodies used to label transporter proteins included a mouse anti- Na^+^/K^+^-ATPase pump (Santa Cruz Biotechnology, sc-48345) and a rabbit polyclonal anti-SGLT2 transporter (Abcam, ab37296). All primary antibodies were incubated in the device overnight at 4°C. All channels were rinsed 3 times with PBS. Secondary antibodies were also mixed in a 1% FBS solution with a 1:1000 dilution of Hoechst. Donkey anti-mouse IgG (AF 488) and anti-rabbit IgG (AF 568) secondary antibodies were incubated in the channels for 1 hour at room temperature. A final rinsing step was done with PBS 3 times before samples were ready to image.

### Cell density, viability and metabolic activity in device

The number of hRPTEC per square mm of cell culture area, both of co-culture and non-co-culture conditions, was determined using the cell analysis software, CellProfiler. A pipeline was written so the cell images with nuclei labeled with Hoechst were inverted, masked and the background smoothed such that each nucleus could be identified and counted in a high throughput, objective manner. Cell viability and metabolic activity was measured for each cell culture condition 24 hours after seeding cells in device, using the WST-1 assay (Millipore, catalog no. 2210) in the same hMVEC-complete medium so as not to affect the final absorbance reading. A 1:10 dilution of the tetrazolium salt solution was made in culture medium and incubated in the device channels for 30 minutes. 100 μl of the 125 μl total volume was collected into a 96 well plate and absorbance of the sample volume was measured at 440 nm with a reference wavelength over 600 nm using a multiwall spectrophotometer. Background absorbance was subtracted to get final absorbance reading, which was normalized to average cell count per square mm.

### Reabsorptive function assay

Reabsorption of the fluorescent glucose analog, 2-NBDG, served as a metric to monitor the transport function of the PT co-culture model in our device. The reabsorption of 2-NBDG from the filtrate/epithelial channel to the vascular/endothelial channel was observed using z-stack microscopy coupled with time-lapse image acquisition. 2-NBDG was infused into the filtrate channel under control (no drug) or sodium transport-inhibited conditions (1 μM ouabain). The alternating use of drug and no-drug serves as an internal control to demonstrate active reabsorption. The starting condition of either no drug or 1 μM ouabain was alternated for each tissue sample tested. A Zeiss LSM 700 confocal microscope was used to capture all images. A 3-slice z-stack captured intensity values of the tubule channel, the hRPTEC tissue layer, and the vascular channel at a rate of 1 slice every 4 seconds. The scan time per frame was 3.87 seconds. The change in fluorescent intensity over time in the vascular channel corresponded to the movement of 2-NBDG into that channel under control, drug, or recovery conditions. All tissue samples were glucose-starved for 30 minutes prior to the experiment in buffer containing 0.5% BSA, 5 mM sodium pyruvate and 5 mM glucose in PBS containing calcium and magnesium. Under drug conditions, 1 μM of ouabain was infused via a syringe pump into the vascular channel until the 2-NBDG signal was insignificant in the vascular channel. The pump was stopped and the reabsorption of 2-NBDG into the vascular channel was observed under a static environment. 2-NBDG solution was regularly replenished to keep the 2-NBDG concentration constant in the filtrate channel. For recovery conditions, the pump was used to rinse ouabain solution from the vascular channel. 2-NBDG solution was replenished in the tubule channel and 2-NBDG reabsorption into the vascular channel was again observed. This method was repeated with a second administration of 1 μM ouabain to further demonstrate the dynamic reabsorptive function of the tissue model.

### Imaging collection and statistics

A Zeiss Axiovert inverted epifluorescent microscope with an attached camera and the image acquisition software, Zen Blue 2012, were used to collect cell images. Exposure time was controlled during each image acquisition when comparing fluorescent intensity expressions. A Zeiss LSM 510 confocal microscope was used to take z-stacks and single images of the co-culture samples. For each experiment, at least 5 images for each tissue layer and at least three replicate samples were analyzed per group. Groups were compared for statistical significance using a one-way ANOVA test with a paired Tukey analysis if multiple groups were being analyzed, or a t-test was used when comparing 2 groups. Variation within each group of data is represented by error bars, defined by s.e.m. The variance was similar between all groups that were statistically compared. The statistical analysis for the 2-NBDG transport considered the full ensemble of data to ensure maximum statistical power and provide a complete picture of the relationships. The data were modeled using an analysis of covariance with time as the covariate. To capture the non-linear behavior of 2-NBDG over time, the model included time and time squared as covariates. Main effects modeled the changes across the four phases (control, initial 1 μM of ouabain, recovery, and second 1μM of ouabain) and mean differences across the three devices. All terms in the analysis of covariance model were highly statistically significant (p-values < 0.0005). Thus, the analysis of covariance model indicates that 2-NBDG intensity in the vascular channel follows a consistent pattern across all devices, with the level rising during the control and recovery periods, but clearly depressed during the two drug (1 μM of ouabain) conditions. Experimental results were verified for 3 independent experiments.

## Results and discussion

### Establishing physiological human renal proximal tubule architecture and barrier function in vitro

Sub-micron surface topography provides important signals to influence cell function *in vitro* [11,12,13,14,15]. We incorporated a porous, topographically-patterned polycarbonate membrane into the microfluidic PT, with 750 nm wide grooves that are within an order of magnitude of the sub-micron feature sizes observed in renal BM [16,17]. The embossed membranes separated a microchannel representing the tubule filtrate lumen from a microchannel representing the microvascular lumen, with the patterned side of the membrane serving as the floor of the epithelial cell chamber as shown in Fig 1C and 1D. The device retained the ability to apply FSS, with levels similar to those found in renal tubules *in vivo* of 0.3–1 dynes/cm^2^ [18,19], although it was employed with the topographical patterns without application of FSS for a significant duration. We have previously shown that the micropatterned topography enhanced formation of tight epithelial cell monolayers compared to cell populations cultured on non-topographical surfaces, in both the presence and absence of FSS [20]. Further characterization of the influence of the topography in the current configuration could be evaluated in future studies.

**Fig 2:**
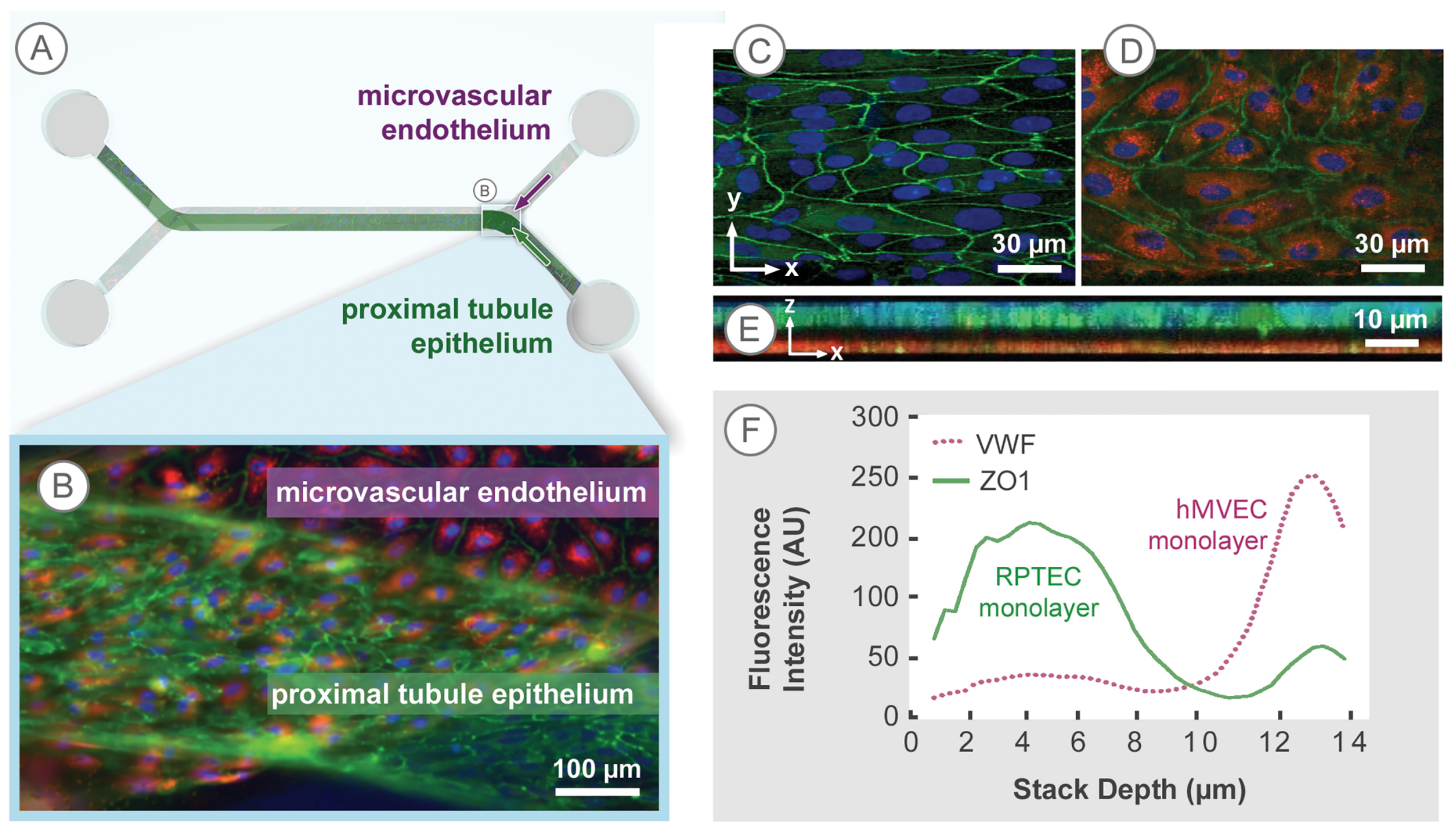
hRPTEC and hMVEC in close-contact co-culture form a physiological PT tissue barrier. (A) The microfluidic channels overlap to create a renal epithelial filtrate channel in communication with an endothelial vascular channel. (B) A close-contact co-culture of hRPTEC and hMVEC were grown in channels on opposite sides of the membrane. hRPTEC were labeled with anti-ZO-1 (green) and hMVEC with anti-vWF (red). (C, D) Confocal slices of the co-cultured cells show a confluent hRPTEC tissue layer and hMVEC tissue layer with clear ZO-1 (green) and vWF (red) expression, respectively. Each tissue layer exists in the xy plane, but is separated in the z-axis by the membrane. Scale bars: 30 μm. (E) A collapsed xz view of the co-culture stack shows clear separation between the tissue layers and a thicker epithelial tissue layer vs. endothelial layer. (F) A z-profile plot illustrates the change in average intensity expression, normalized to respective blank channel intensity values, of ZO-1 and vWF signals through the 14 μm co-culture 3D tissue stack. The width of the peaks correspond to each cell layer thickness, indicating a cuboidal morphology of the hRPTEC tissue in and a squamous morphology of hMVEC tissue. At least 3 replicate samples were repeated over at least 3 batches of experiments.

One channel of the microfluidic device overlaps with the other to create a renal epithelial filtrate channel in communication with an endothelial vascular channel, shown in Figs 1B and 2A. hRPTEC and hMVEC formed confluent tissue layers on either side of the topographically patterned membrane, shown in Fig 2B. The TJ protein, ZO-1, was expressed in the hRPTEC layer as shown in Fig 2C, while the hMVEC layer expressed the endothelial-specific marker von Willebrand Factor (vWF) as shown in Fig 2D. Each tissue layer covered the width of the channel without exhibiting edge effects at the channel walls. Fig 2E shows a collapsed xz plane view, illustrating the hRPTEC layer (green) with a more cuboidal morphology, mimicking the *in vivo* state [21], compared to the thinner hMVEC layer (red), reflecting the expected squamous-like morphology. The non-colored interspace indicated the location of the porous membrane. The average intensity of each tissue slice for a given fluorescent channel was quantified in a z-profile intensity plot profile shown in Fig 2F. The tissue stack spanned 14 μm, starting with the hRPTEC tissue layer at 0–8 μm and ending with the hMVEC tissue layer at 11–14 μm. The membrane, indicated by the drop in intensity at 8–11 μm, clearly separated the two tissues. The width of the 2 peaks was indicative of cuboidal and squamous morphologies of the respective epithelial and endothelial cells, which corresponds with the cross section seen in Fig 2E. Thus, the co-culture architecture mimicked the physiological structure of a renal proximal tubule, placing the basolateral sides of hRPTEC and hMVEC layers in close proximity, separated by the ECM-coated porous membrane. The tissue structure and protein expression illustrated by the confocal microscope z-stacks indicated formation of tissue layers with polarized renal morphology. This morphology allowed study of renal-specific transport function across both tissue layers, similar to the complete pathway solute and water take to transfer from the tubule lumen to the microvascular space *in vivo*.

### hRPTEC monolayer formation and activity are enhanced in the presence of hMVEC

**Fig 3:**
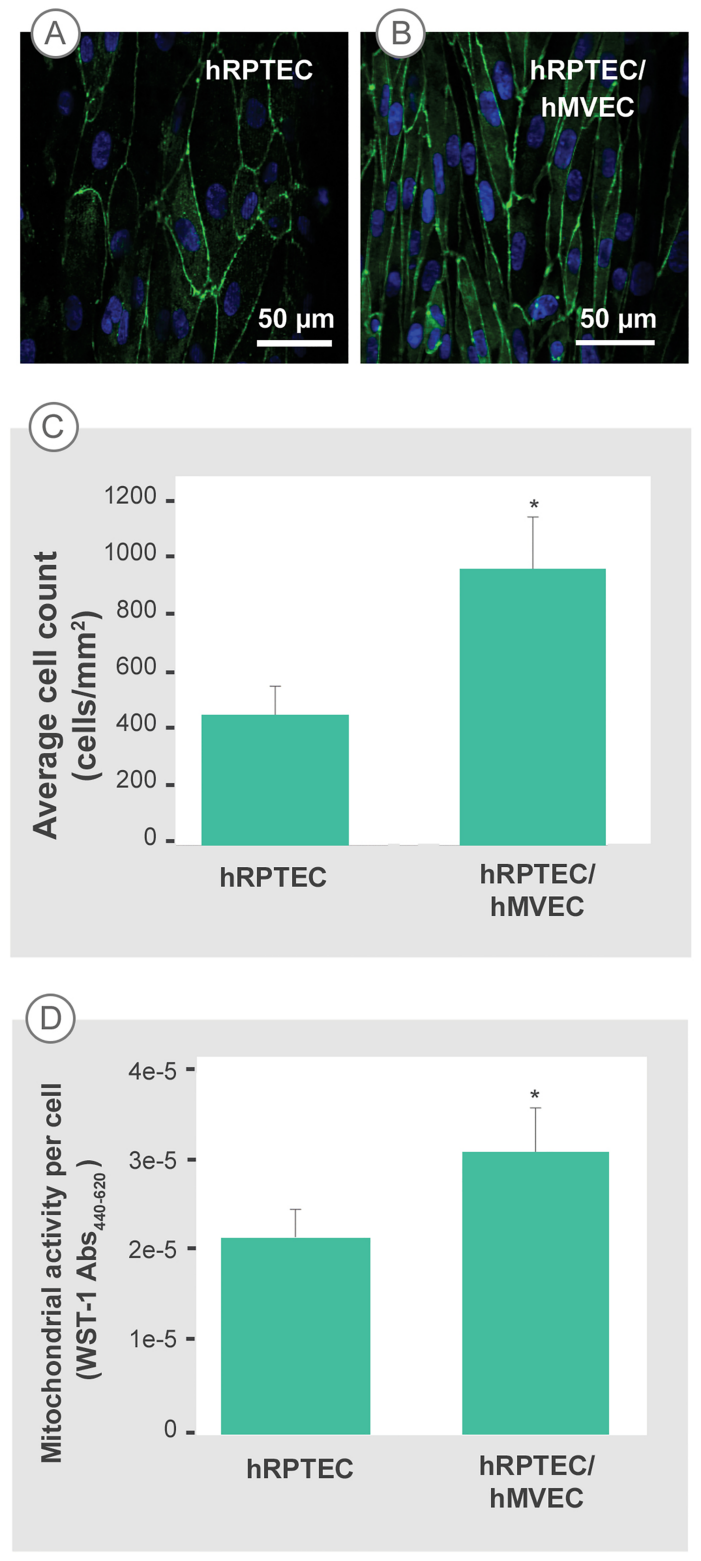
hMVEC presence enhances the hRPTEC layer. (A, B) TJ formation in 7-day cultures of hRPTEC without and with hMVEC in the microfluidic device, respectively. hRPTEC formed a more compact tissue layer with clear TJ formation under hRPTEC/hMVEC co-culture conditions. hRPTEC were labeled with anti-ZO-1 (green) and Hoechst (blue). At least 5 images for each tissue layer and at least three replicate samples were analyzed per group. (C) Average number of hRPTEC/mm^2^ in co-culture conditions is more than double that of hRPTEC-only conditions after 7 days of culture. * P < 0.001. Results were verified for 3 independent experiments. (D) hRPTEC in co-culture conditions have increased mitochondrial activity compared to hRPTEC-only conditions, normalized to cell count. The mitochondrial activity of hMVEC cells in co-culture is negligible. * P = 0.002. Error bars represent standard error of the mean from 3 independent tissue samples.

hRPTEC monolayer formation in the microfluidic PT model was highly influenced by the presence of the hMVEC. hRPTEC cells grown in device channels without the presence of hMVEC cells stop proliferating and lose their phenotype after 3 days of culture. Fig 3A and 3B illustrates the tissue layer structural differences between hRPTEC monoculture and hRPTEC/hMVEC co-culture conditions. Over a 7-day culture period, hRPTEC tissue layers were more compact with a higher cell count per surface area and better-developed TJs (green) under co-culture conditions compared to hRPTEC-only conditions. hRPTEC tissue layers formed in mono-culture conditions had 43% less cells per surface area when cultured over the same time period as co-culture conditions, as shown in Fig 3C. hRPTEC tissue layers cultured in mono-culture conditions using the hMVEC media did not survive to form monolayers and were therefore not included in the analysis. Mitochondrial activity of the hRPTEC, measured using the WST-1 bioassay, was enhanced by the presence of hMVEC as shown in Fig 3D. The absorbance signal indicating mitochondrial activity was normalized to the average cell number/mm^2^. The contribution of mitochondrial activity from the hMVEC tissue was negligible, data not shown. The addition of hMVEC tissue into the model also sustained hRPTEC tissue viability for at least 15 days without requiring additional channel perfusion or FSS application.

Heterotypic cell-cell communication present in co-culture environments influences cellular functions including proliferation [22,23], differentiation [24,25], and tissue structure formation [26,27,28,29,30]. In the proximal tubule, because reabsorption of water and solutes occurs through epithelial and endothelial monolayers, both cells types were incorporated into our device to mimic the orientation, separation, and composition of the co-culture found *in vivo*. We speculate that the hMVEC tissue layers were secreting multiple soluble factors, which can have an effect on proximal tubule tissue development[31]. It was expected that cell types, such as hRPTEC and hMVEC, which exist in close proximity in the body, will influence each other *in vitro*, which was consistent with our data of a more compact hRPTEC tissue layer, enhanced hRPTEC proliferation and increased hRPTEC mitochondrial activity when in co-culture with hMVEC. The higher mitochondrial activity per cell, whether due to increase in mitochondria number or individual mitochondria efficiency, may also influence or indicate presence of energy-dependent functions such as active transport[32].

Very few models have been developed to incorporate microvascular tissue to mimic reabsorptive barriers *in vitro [33,34]*. Transwells provide either a non-contact orientation with a 1 mm distance between the two cell types, or cells on both sides of the Transwell insert membrane to establish an approximation of a close-contact orientation of the two cell types[35,36]. The second orientation provides a more realistic architecture, but the Transwells deprive cells of physiological cues [19,37], such as BM topography and FSS which are present *in vivo* and accommodated in our model.

**Fig 4:**
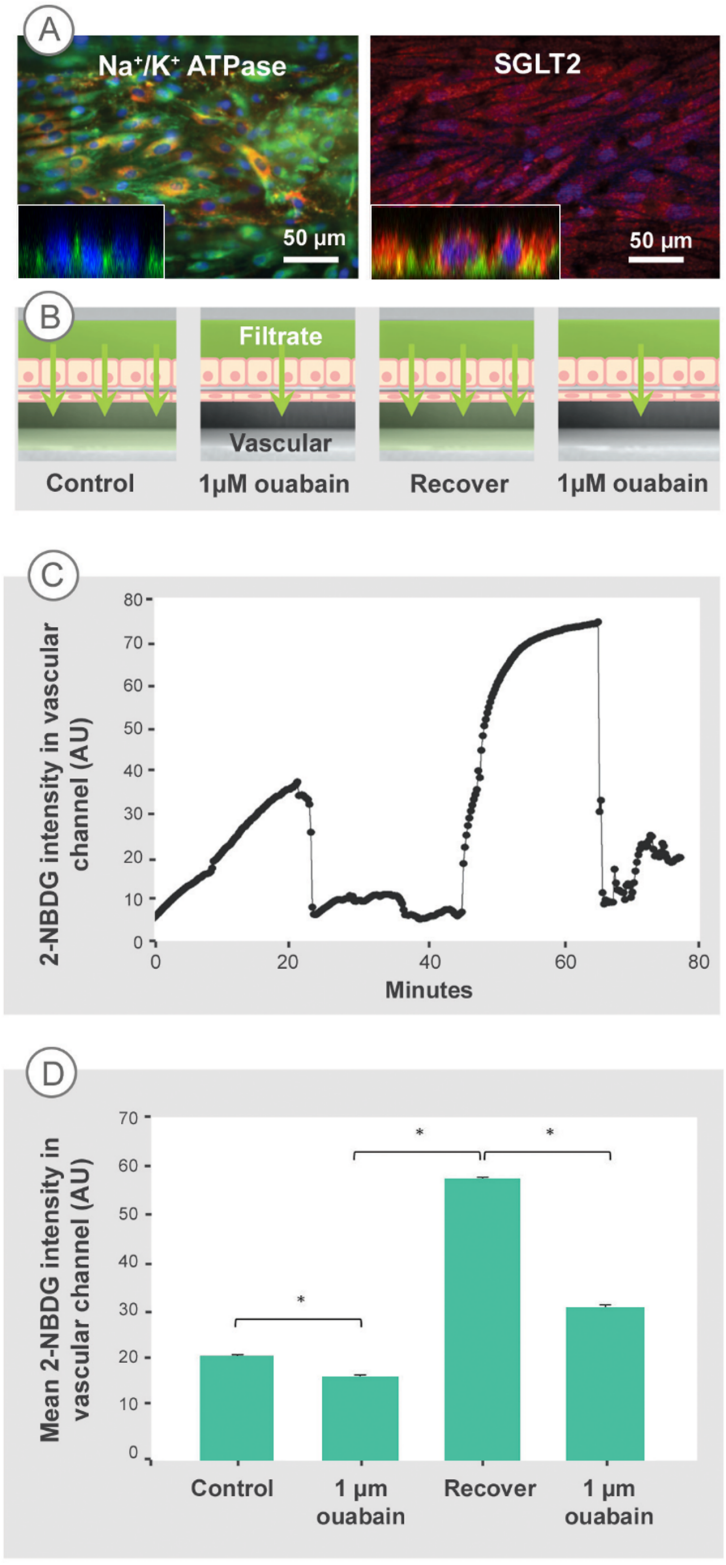
The microfluidic PT model altered sodium-dependent reabsorption of glucose analog in response to ouabain. (A) hRPTEC expressed polarized transport proteins Na^+^/K^+^ ATPase and SGLT2 under co-culture conditions. (B) A schematic representation of experimental conditions. Under all conditions, the transport of a fluorescent glucose analog, 2-NBDG, from the filtrate channel (top) into the vascular channel (bottom) was observed using confocal z-stack and time-lapse microscopy to quantify intensity in the vascular channel. (C) 2-NBDG intensity versus time for one device subjected to all conditions sequentially shows the reduction in active transport due to ouabain administration. (D) The bar graph indicates the mean 2-NBDG intensity in the vascular channel at each condition, measured over ~15 minutes for 3 devices. Data are from 3 replicates of the experiment. Error bars represent standard error of the main effects as computed from the error term in the analysis of covariance model. Introducing ouabain to the vascular channel blocked 2-NBDG transport. The 2-NBDG transport recovered when ouabain was rinsed from the system. A second administration of ouabain again blocked 2-NBDG transport. The tissue recovery and repeat effect of ouabain demonstrates dynamic reabsorptive cell-mediated reabsorption function. * P < 0.0005. Experimental results were verified for 3 independent experiments.

To demonstrate and directly quantify this active reabsorption function in the microfluidic PT, we used the fluorescent glucose analog 2-NBDG[38] to monitor reabsorption in the absence and presence of the Na^+^/K^+^-ATPase inhibitor ouabain [39,40,41]. 2-NBDG is transported by SGLT2 and GLUT2 in cultured kidney cells [42,43,44], and its transport has been directly quantified by fluorescent microscopy [38,45]. The hRPTEC in the device expressed both the Na^+^/K^+^-ATPase localized to the basolateral domain and SGLT2 localized to the apical domain, shown in the IF stained images and inset confocal cross sections in Fig 4A. The device enables the direct quantification of morphology and transport in real time, since its architecture allows a high magnification/low working distance objective and a low volume of fluid to minimize dilution of the transported species. These two features enable precise evaluation of concentration, which would not be possible in either a larger volume Transwell system or would be difficult in 3D microfluidic systems with a non-planar, round channel geometries[45]. In contrast to a proximal tubule model with a round cross sectional area[45], the two-channel microfluidic architecture used here enables physiological delivery of glucose or glucose analog via the filtrate channel and transport-inhibiting drugs via the vascular channel as would be the case *in vivo*. In addition, microfluidic delivery of 2-NBDG, ouabain and a placebo can be controlled independently, which allows for sequential administration of experimental conditions, rather than being limited to a comparison to separate experimental controls. Furthermore, the device architecture ensures quick evaluation of monolayer quality before, during and after experimentation since the tissue layer can be directly imaged in the same focal plane with a 40x objective. Transport of 2-NBDG from the filtrate channel to vascular channel, represented in an experimental schematic shown in Fig 4B, was examined over 4 sequential conditions: 1) control without ouabain; 2) 1 μM ouabain; 3) recovery of reabsorption after rinsing ouabain out from the vascular channel, and 4) repeat dosing of 1 μM ouabain. The alternation of ouabain and control without ouabain served as an internal control. The transport of 2-NBDG from the filtrate channel to the vascular channel of the microfluidic PT was quantified by plotting 2-NBDG concentrations in the vascular channel as it was sequentially exposed to the 4 conditions. An example plot from one microfluidic PT is shown in Fig 4C. The 2-NBDG concentration in the vascular channel increased approximately 8-fold over 20 minutes under control conditions as 2-NBDG was transported from the filtrate to vascular channel. Perfusion of 1 μM ouabain through the vascular channel decreased the 2-NBDG concentrations as 2-NBDG was flushed from the channel for 10 minutes. After perfusion of ouabain, little to no transport of 2-NBDG was observed in the vascular channel. Once ouabain was rinsed from the system, 2-NBDG concentrations in the vascular channel sharply increased over 15 minutes, indicating recovery of tissue transport function. A repeat rinsing with and dosing of 1 μM ouabain resulted in a marked reduction of 2-NBDG transport compared to the 2-NBDG transport in recovery or control conditions. As a measure of transport rate, Fig 4D plots the mean 2-NBDG concentration in the vascular channel over 15 minutes of each experimental condition for three independent replications of the experiment. The grouped data corroborated the dynamic reabsorptive function exhibited in the time plot. Transport of 2-NBDG recovered to control levels or higher when ouabain was rinsed from the microfluidic PT. A repeat dose of ouabain induced a second decrease in reabsorption to less than half of the average of the recovery concentration. This measure of transport indicates significantly different reabsorption of the glucose analog due to presence of ouabain. The dynamically-responsive transport of 2-NBDG in response to ouabain indicated active reabsorptive function across intact, polarized epithelial and endothelial cell monolayers in the microfluidic PT device.

*In vitro* models of kidney tissue are commonly characterized by the expression of transport proteins [46], secretion of factors [47], and the uptake, rather than reabsorption, of solutes such as glucose and sodium [44]. These approaches imply functional properties of *in vivo* kidney tissue, but do not directly demonstrate those functions, the most important of which is reabsorption. Our model configures the tissue layer to allow the reabsorption to occur across a complete epithelial-to-endothelial barrier, while directly quantifying the active reabsorption function [48]. The optical access in our model ensures the evaluation of morphology and expression, while adding a direct measurement of active kidney function and maintaining the ability to evaluate secretion or uptake.

Engineered structural features enabled the microfluidic PT to demonstrate the major function of the proximal tubule: reabsorption of water and solute. This active solute reabsorption depends on parameters such as formation of tight epithelial and endothelial monolayers, and polarized expression of transporters[48]. We demonstrated this function, and consequently its dependent parameters, by direct measurement of sodium-coupled glucose transport from the filtrate channel to the microvascular channel. Immunofluorescent labels demonstrated expression of the epithelial Na^+^/K^+^-ATPase pump and glucose transporter SGLT2 in our co-culture system. Application of the fluorescent glucose analog, 2-NBDG, in the filtrate channel led to an increase in fluorescence intensity in the vascular compartment. This transcellular movement of glucose required active sodium transport, since inhibiting the sodium pump with ouabain delivered through the vascular channel led to ~85% reduction in glucose reabsorption within 15 minutes. The effect was reversed when the drug was rinsed away from the epithelial channel, and was repeated when the drug was introduced a second time. Ouabain binds with high affinity to the Na^+^/K^+^-ATPase pump in normal kidney cells and it was expected that 1 μM would inhibit at least 70% of the pump’s activity [40], which is similar to the 85% reduction of glucose reabsorption seen in our devices. The recovery of reabsorptive function and repeat decrease of 2-NBDG transport in response to ouabain in our kidney model confirms the alterations in transport of 2-NBDG are due to the drug acting on transport proteins of the transcellular pathway and not to changes in TJ permeability or in other paracellular pathways. It is unknown if the endothelial layer influences the reabsorptive function of the epithelial layer, although we saw that in non-co-culture conditions, the epithelial monolayer was inconsistent and difficult to maintain more than 2–3 days in the device. Moreover, both layers are needed to recapitulate the *in vivo* transport pathway, which necessitated their simultaneous use here. This is a clear demonstration of sodium-coupled glucose transport in a proximal tubule-like device, in contrast to previous models, which do not demonstrate transport directly [42,49,50]. The presence of this complex reabsorptive function in our microfluidic model is significant because it demonstrates the ability of an *in vitro* system to recapitulate a major function of the proximal tubule, in addition to replicating physiological architecture and molecular uptake.

## Conclusion

In conclusion, we have engineered a proximal tubule-like tissue in a microfluidic device, which copies major structural, morphologic, and cellular components of the *in vivo* proximal tubule. Our microfluidic PT platform has two key structural features engineered to mimic normal PT tissue architecture, which distinguish it from previous models. First, the incorporation of endothelial-epithelial co-culture while leveraging microfluidic architecture, and second, the device architecture allows direct monitoring and measurement of cellular morphology and functional responses in real time. Importantly, the engineered microfluidic PT replicates the kidney-specific active function of reabsorption essential to this nephron segment. Such a device could be linked to proximal and distal nephron components, each populated by specialized cells providing a basis for a kidney rebuilding strategy. The current device is set up for future investigation of the effects of sub-micron surface topography on epithelial and endothelial function in this coculture system, assessment of additional transporter function, including organic anion and cation transport, and evaluation of the influence of FSS in one or both tissue compartments. The microfluidic PT can reconstruct human kidney physiology and function at the cellular and tissue level, screen new drugs for potential kidney toxicity without the need for animal models, elucidate the mechanism of action of kidney-acting drugs, and be scaled to generate a clinically-relevant construct or device with proven kidney function.
